# How Neurophysiological Measures Can be Used to Enhance the Evaluation of Remote Tower Solutions

**DOI:** 10.3389/fnhum.2019.00303

**Published:** 2019-09-06

**Authors:** Pietro Aricò, Maxime Reynal, Gianluca Di Flumeri, Gianluca Borghini, Nicolina Sciaraffa, Jean-Paul Imbert, Christophe Hurter, Michela Terenzi, Ana Ferreira, Simone Pozzi, Viviana Betti, Matteo Marucci, Alexandru C. Telea, Fabio Babiloni

**Affiliations:** ^1^Department of Molecular Medicine, “Sapienza” University of Rome, Rome, Italy; ^2^BrainSigns srl, Rome, Italy; ^3^IRCCS Fondazione Santa Lucia, Neuroelectrical Imaging and BCI Lab, Rome, Italy; ^4^French Civil Aviation University (ENAC), University of Toulouse, Toulouse, France; ^5^Department of Anatomical, Histological, Forensic & Orthopedic Sciences, “Sapienza” University of Rome, Rome, Italy; ^6^DeepBlue srl, Rome, Italy; ^7^Department of Psychology, “Sapienza” University of Rome, Rome, Italy; ^8^Braintrends Limited, Applied Neuroscience, Rome, Italy; ^9^Department of Mathematics and Computing Science, University of Groningen, Groningen, Netherlands; ^10^College Computer Science and Technology, Hangzhou Dianzi University, Hangzhou, China

**Keywords:** EEG, ECG, GSR, eye blink, mental workload, remote tower air traffic management, machine learning, asSWLDA

## Abstract

New solutions in operational environments are often, among objective measurements, evaluated by using subjective assessment and judgment from experts. Anyhow, it has been demonstrated that subjective measures suffer from poor resolution due to a high intra and inter-operator variability. Also, performance measures, if available, could provide just partial information, since an operator could achieve the same performance but experiencing a different workload. In this study, we aimed to demonstrate: (i) the higher resolution of neurophysiological measures in comparison to subjective ones; and (ii) how the simultaneous employment of neurophysiological measures and behavioral ones could allow a holistic assessment of operational tools. In this regard, we tested the effectiveness of an electroencephalography (EEG)-based neurophysiological index (W_EEG_ index) in comparing two different solutions (i.e., Normal and Augmented) in terms of experienced workload. In this regard, 16 professional air traffic controllers (ATCOs) have been asked to perform two operational scenarios. Galvanic Skin Response (GSR) has also been recorded to evaluate the level of arousal (i.e., operator involvement) during the two scenarios execution. NASA-TLX questionnaire has been used to evaluate the perceived workload, and an expert was asked to assess performance achieved by the ATCOs. Finally, reaction times on specific operational events relevant for the assessment of the two solutions, have also been collected. Results highlighted that the Augmented solution induced a local increase in subjects performance (Reaction times). At the same time, this solution induced an increase in the workload experienced by the participants (W_EEG_). Anyhow, this increase is still acceptable, since it did not negatively impact the performance and has to be intended only as a consequence of the higher engagement of the ATCOs. This behavioral effect is totally in line with physiological results obtained in terms of arousal (GSR), that increased during the scenario with augmentation. Subjective measures (NASA-TLX) did not highlight any significant variation in perceived workload. These results suggest that neurophysiological measure provide additional information than behavioral and subjective ones, even at a level of few seconds, and its employment during the pre-operational activities (e.g., design process) could allow a more holistic and accurate evaluation of new solutions.

## Introduction

There are many small airports all around the world. For these airports, it is more and more difficult to maintain the cost-effectiveness of the operations. In order to improve cost-efficiency in air traffic provision, many countries are currently considering the remote tower air traffic control. The general idea is that the air traffic controller (ATCO) does not have to be located in the tower of the aerodrome that he is controlling, but he can operate from another location. With respect to standard control towers, this new solution will allow monitoring the traffic in small airports thanks to high-resolution cameras, advanced sensors and radio transmissions. The idea is that ATCOs should continuously interact with the tools that they are currently using but in a room that has a video wall that emulates the outside of a control tower. Anyhow, some implied information which could be crucial for ATCOs at a specific moment could be lost in a remote tower environment. For this reason, in the last years many tools have been developed to try to enhance the performance of the operator, by providing synthetic feedback able to replicate, and even to augment real tower sensations (i.e., vibrations and/or sounds from the surroundings (Reynal et al., 2019). In this regard, Augmented Reality is nowadays one of the most important enabling technologies for innovation in a number of operational environments, since it offers the possibility to enhance the user interaction with the real environment by adding information to it in the form of synthetic overlays that enhance the user perception of the surrounding world (Masotti and Persiani, 2016). Dealing with an exponential increase of novel technologies and solutions able to enhance operator performance, it becomes essential to have reliable and precise tools to support the design phase.

New technologies or novel solutions in operational environments are often, among objective measurements, evaluated by using subjective assessment and/or judgment from operational experts (Fürstenau et al., 2009; van Schaik et al., 2010; Papenfuss and Möhlenbrink, 2016). However, it has been widely demonstrated how such kind of measures could suffer from poor resolution due to a high intra and inter-operator variability depending on the nature of the measure itself (i.e., subjective). In addition, it is widely accepted in scientific literature the limit in using subjective measures alone, such as questionnaires and interview, because of the impossibility to quantify “unconscious” phenomena underlying human behaviors (Gopher and Braune, 1984). Performance measure, when available, could provide just a part of the story, since an operator could achieve the same performance level by using different solutions, but experiencing different cognitive resources (e.g., experienced workload).

In this regard, in the last decade it has been demonstrated that neurophysiological measures could be used to assess human mental states, and that such kind of measures would achieve a higher resolution with respect to subjective assessing measures, providing additional information with respect to performance-based ones (Aricò et al., 2016a; Blankertz et al., 2016). With respect to subjective measures, the neurophysiological ones have the advantages to not impact on the task performed by the user, since they do not require any explicit input. Moreover, they can even be measured online, i.e., during the execution of the task (Parasuraman, 2001; Gevins and Smith, 2003). In this context, mental workload is one of the most studied human mental states, because of its strong connection with the user’s performance variation, affecting human error, system safety, productivity and operator satisfaction (Xie and Salvendy, 2000). The evaluation of mental workload by using (only) subjective measures is a quite disputed point in scientific literature. de Winter (2014) stated that “*mental workload is an operational concept and not a representational concept, the idea that mental workload can be captured by the use of a questionnaire, and in particular by the use of the NASA-TLX alone, is too simplistic*.” Mental workload is a more complex dynamic concept that needs to be assessed by more than just ratings on a subjective scale (de Waard and Lewis-Evans, 2014). Mental workload reflects available cognitive resources (i.e., attentional resources and working memory capacity) during the execution of a task (de Waard and Lewis-Evans, 2014). It is widely demonstrated in literature as different biosignals features which are quite sensitive to workload variations. For example, eyes blinks, the rapid closing and reopening of the eyelid, is considered to be an indicator of both fatigue and workload. Recarte et al. (2008) suggested that the specific amount of visual attention required by the task could lead to a blink inhibition but that the fatigue associated with long tasks would impair such inhibition. The monitoring of Heart Rate (HR) is commonly reported as being a measure sensitive to variations in mental workload. In particular, an increase in workload induces an increasing in HR (Jorna, 1993). Regarding brain activity, electroencephalography (EEG) represents one of the most widely used techniques to infer relevant information regarding workload variations, because of its higher temporal resolution and relatively lower cost with respect to other brain imaging techniques (e.g., fMRI, MEG). One of the most studied features related to workload variation are frontal theta and parietal alpha bands. Activation of frontal and right parietal cerebral regions, reflected by a synchronization in the theta band (4–8 Hz) and a desynchronization in the alpha band (8–12 Hz), is sensitive to working memory load (Klimesch, 1999). In the same way, increase in attentional resources has been linked to a desynchronization of the alpha band (Klimesch et al., 1998) and theta band synchronization (Gevins and Smith, 2000). Both working memory and attention share the same cerebral regions and vary in the same way for numerous tasks. Anyhow, alpha-band synchronizations were also found during tasks soliciting frequent task switching (Pope et al., 1995). Other works highlighted an increase in alpha band power with an increase in task demand, especially as the number of tasks increased (Kamzanova et al., 2014). Also, it was proposed that both alpha-band synchronization and desynchronization might be responsible for two different working memory maintenance mechanisms (Capilla et al., 2014). As a result, alpha-band synchronization would support interfering item inhibition while alpha-band desynchronization would support relevant item maintenance (Puma et al., 2018). Moreover, the cognitive psychology literature demonstrates that the human psychophysiological activation, i.e., the arousal, has an “inverted U-shape” relationship with performance in that some levels of activation may help an individual to perform at a level that is higher than their baseline state (Yerkes and Dodson, 1908). Although this theory has been in some ways corrected and revised (Landers, 1980; Arent and Landers, 2003), the propaedeutic role of a proper psychophysiological activation to achieve the own best performance is a pillar of the behavioral researches (Eysenck, 1982). In this field, the skin sweating has been demonstrated to be one of the most sensitive physiological reactions to arousal variations (Bach et al., 2010), therefore the galvanic skin response (GSR) is considered the gold-standard bioindicator of human arousal (Boucsein, 2012).

Although many works have demonstrated the possibility to measure user’s mental states in laboratory settings by using neurophysiological measures, just few works demonstrated the applicability of neurophysiological measures out of the labs i.e., real/realistic settings (Aloise et al., 2013; Cartocci et al., 2015; Aricò et al., 2016a, 2017a, 2018b; Blankertz et al., 2016; Vecchiato et al., 2016; Borghini et al., 2017a, b; Cartocci et al., 2018a,b, 2019a,b; Di Flumeri et al., 2018; Modica et al., 2017, 2018). Even less, the studies demonstrating the effectiveness of such measures in comparing different solutions in operational environments. In fact, although the recent technological achievements in wearable devices research towards the possibility to measure biosignals (e.g., brain, heart and ocular activities) with a zero invasiveness for their employment in operational activities (Di Flumeri et al., 2019), the technology, especially regarding EEG sensors, is not still ready for this kind of use (see Aricò et al., 2017a,b, 2018a). On the contrary, pre-operational activities (e.g., designs of new solutions) could already benefit of this technology, since requirements in terms of invasiveness could be lower, benefiting instead from a powerful user’s evaluation technique (i.e., neurophysiological measures).

In this regard, Borghini et al. (2015) performed a pilot study on professional helicopter pilots performing simulated missions carried out at AgustaWestland facilities in Yeovil (UK) with the aim to compare different avionic technologies not only in terms of performance, but also regarding the experienced workload during their use by using an EEG-based index. Aricò et al. (2018b) proposed a work aiming at investigating the possibility of employing neurophysiological-based measures to assess the human-machine interaction effectiveness. In particular, two different interaction modalities (normal and augmented) related to the Air Traffic Management (ATM) field have been compared in terms of behavioral performance and an EEG-based workload index (frontal theta and parietal alpha ratio), by involving professional ATCOs in a control tower simulated environment at ENAC, Toulouse (France) in a laboratory-based task.

### Research Questions

In the current study, we demonstrated: (i) the effectiveness of neurophysiological measures in comparison with subjective ones; and (ii) how the simultaneous employment of information coming from neurophysiological measures and behavioral ones could allow a holistic comparison of different solutions in remote tower pre-operational activities (i.e., design testing of new operational solutions).

In this regard, we employed specific neurophysiological, subjective and behavioral measures during: (i) the execution of two operational scenarios in remote tower operations; and (ii) specific critical events within each scenario, with the aim to compare the two investigated solutions.

## Materials and Methods

### Experimental Platform

**Figure 1:**
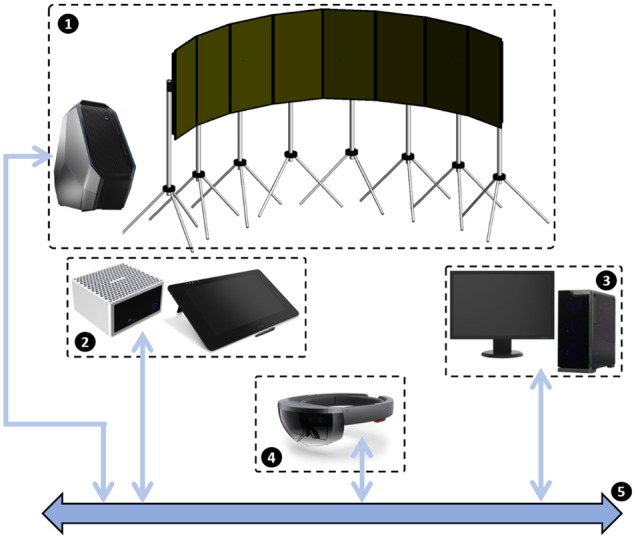
The general setup used for the experiment. (1) The panoramic view to display airport vicinity using eight UHD screens and the Alienware Area51 computer (2) and (3) Computers and screens to display respectively ground and air radars (4) Microsoft HoloLens device to retrieve user’s head movements (5) ENAC Ivy bus software to make all these equipment communicate together.

The Simulation Platform used for the experiment has been developed by using ENAC facilities (Figure 1). The platform was designed to be able to simulate with high realism a Remote Tower environment. In this ecological perspective, the visuals on the airport vicinity had to be as realistic as possible. Therefore, we used a photorealistic view of Muret, an airport located in the South of France and under the supervision of the French Directorate General for Civil Aviation. The view was composed of several photographs of the airport previously stitched together and managed by an *ad hoc* software made with Unity and called RealTower.

To display the out-of-the-window view on Muret airport (RealTower software), we used an Alienware Area51 computer equipped with an Intel Core i7 processor and two Nvidia GeForce GTX 1080 graphic cards coupled together with SLI technology. This computer was connected to eight UHD screen (Iiyama Prolite X4071). Spatial sound was relayed using the speakers from these Iiyama monitors. The choice to use the screen’s speakers instead of binaural sound made with Head Related Transfer Functions, for example, was justified with the fact that we had to have a simple way to promote the platform to several people through demo sessions. The air radar view was managed using a Dell Precision 5810 computer equipped with an Intel Xeon processor and a Nvidia Quadro M500 graphic card. The ground radar view was displayed using a Zotac Magnus EN980 computer equipped with an Intel Core i5 processor and a Nvidia GeForce GTX 980 graphic card.

**Figure 2:**
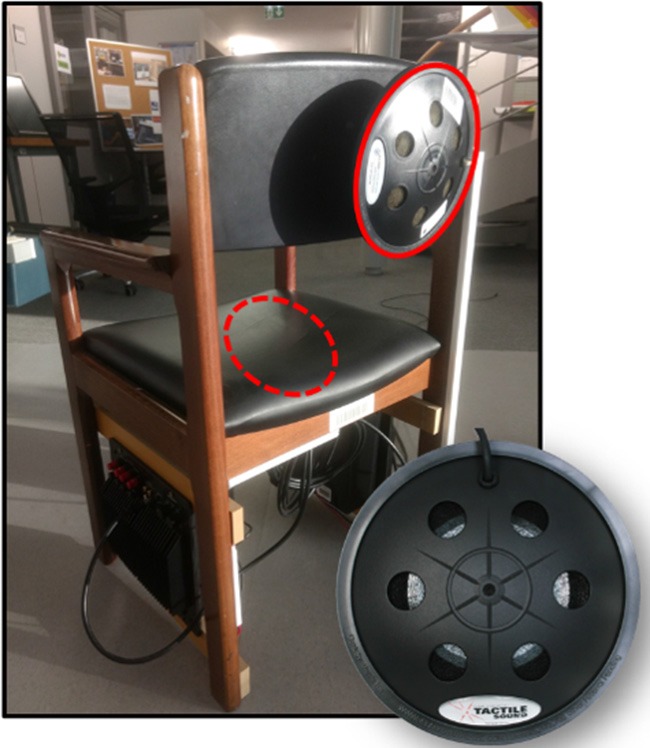
The wooden chair on which two tactile transducers have been attached to spread vibrotactile feedback (see the two red circles).

A homemade version of a chair was also used to spread vibrotactile feedback to the user (see Figure 2). Two Clark Synthesis T239 Gold tactile transducers have been attached on it, one behind the back and another one under the sit. All this equipment communicated with each other *via* software, written in C#, Java and Python, and connected through an *ad hoc* bus called Ivy (Buisson et al., 2002), which makes it possible to easily transmit network messages by means of mechanisms based on regular expressions.

**Figure 3:**
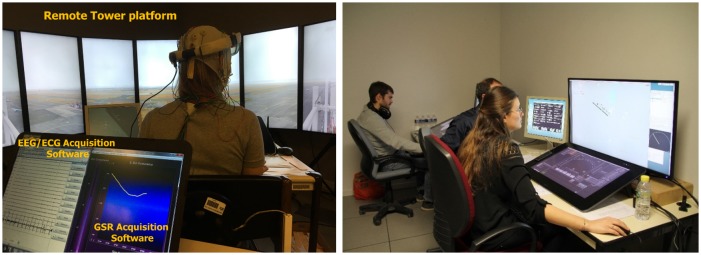
Experimental setup (left) and pseudo-pilots positions used during the experiment (right). Pseudo-pilots have given written informed consent for the publication of this image.

Because of the electromagnetic field generated by the haptic chair’s transducers, no inertial unit could be used. Hence, we chose to monitor participants’ head movements using a Microsoft HoloLens headset device, without using its augmented reality features (Figure 3).

Besides, two pilot positions (called “pseudo-pilot” positions) have been designed to simulate real flight communications (see Figure 3). These facilities were composed of two ground radars and communication means (e.g., microphones working in network coupled with two Griffin Powermate push-to-talk buttons) to make the pseudo-pilots able to dialog in real-time with the subject ATCO during the experiment.

### Experimental Task and Working Scenarios

The experimental task consisted of two different scenarios of 10 min long. In one case, the scenario was supported by novel augmented solutions (*augm-RT*). For the other scenario, it was not provided any augmentation solution, so it had to be performed in a standard operational way (*norm-RT*).

These scenarios were designed with the help of two professional ATCOs, with the goal to be as much realistic as possible. Specific operational events that ATCOs often encounter during their working time have been enclosed in such scenarios.

Therefore, the two scenarios were similar from an operational point of view (i.e., same kind and number of operational events) and overall difficulty level, but not exactly the same, to avoid any habituation and/or expectation effect of the experimental subjects. To avoid any confounding due to eventual differences in the two scenarios, augmented solutions were applied for half of the experimental group on Scenario 1 (and Scenario 2 was without augmented solutions), and the other half on Scenario 2 (and Scenario 1 was without augmented solutions). Scenarios were run by two pseudo-pilots placed in a different room from the one where the experiment was conducted.

Here below they have been summarized the schedule of each scenario:

–*Scenario 1*: four aircrafts are parked at the Muret—Lherm Aerodrome at the beginning of the scenario. Another aircraft is scripted to be in downwind position 3 min after the start approximately.

–*Scenario 2*: four aircrafts are parked at the Muret—Lherm Aerodrome at the beginning of the scenario. Another aircraft is scripted to be in downwind position at 3 min 30 s approximately, and another one is scripted to be in downwind position at 9 min 30 s approximately.

In the two scenarios, one SPATIAL event and two RWY events are scripted to have a minimum number of recordings. However, pseudo-pilots were encouraged to raise more events during the experiment. Therefore, in the recordings, we always had more than one SPATIAL and two RWY events per participant. For the analysis, we always considered the same number of events to compare the two scenarios (*norm-RT* and *augm-RT*).

In addition to the operational scenarios, we designed two more scenarios at two different difficulty levels, to be used to calibrate the W_EEG_ algorithm (see “Neurophysiological Measures” section for further details). These two scenarios were designed by using the same operational characteristics of the two experimental scenarios, e.g., the ATCO point of view, visibility conditions, type of task required (i.e., remote tower operations). They were simulated pilots asking to start their engine, then to reach the holding point, before finally requesting a take-off, while other ones were aligned for landing. One scenario was composed of a few number (i.e., two) of aircrafts (*EASY*), and the other one integrated a high number of aircrafts (i.e., eight) in order to increase the difficulty level of the required task (*HARD*). Also, within *EASY* scenario, only three actions were required from pseudo-pilots, while within HARD scenario, there were 17 different actions requested.

Once the experimental platform and experimental task were ready, a shakedown test with three professional ATCOs has been conducted in order to ensure that the components of the platform and the augmented solutions were working properly and that the experimental setup achieved the maximum level of operability and realism.

From an operational point of view, we expected that the proposed Augmented solutions should be able to enhance performance and/or induce a reduction in cognitive workload experienced by the operators with respect to standard operation (i.e., no augmented solutions activated).

### Design and Rationale for Augmentation Modalities

Specific events which could occur on the airport are crucial in terms of safety and therefore are of particular interest in the ATM domain and have been taken into account during the design phase of the experimental scenarios. These events are reported below:

- *Unauthorized movements on ground*: it occurs when an aircraft starts its engine and starts to move on the parking area or the taxi circuit without previously asking for it or simply warn the control tour;
- *Runway incursion*: it represents one of the most dangerous events that could occur on the runway, it consists in an aircraft entering the runway without permission while another one is about to land soon (i.e., on its final approach segments).

These events were enclosed in both scenarios the same number of times. Two specific augmentation modalities (i.e., a distinct one for each of these two events) have been designed to give the ATCOs a way to solve these events faster while being more aware of the highlighted situations. The following sections describe these two interaction modalities and related feedback to the operator.

#### Spatial Sound Alert for Unauthorized Event on Ground

Spatial sound alert modality is used to warn the participant that an unauthorized event on ground (unauthorized start of aircraft engines or taxiing procedure) has been detected. The goal is to keep the participant’s attention toward the abnormal event. This related event is called “SPATIAL” in the present article. The head of the participant is constantly tracked using Microsoft HoloLens device to monitor whether his/her gaze is actually aligned with the related event or not. Spatial sound alert modality uses an audio cue. A distinctive sound is played in order to unequivocally attract the participant. Basically, this sound is composed of three pure sine waves at 880 Hz (A) of 50 ms length separated by 50 ms of silence, looped continuously until the participant’s head is aligned with the event. To avoid creating doubt in case the participant would have anticipated the situation and is already focused on, the alert is not activated if the gaze of the participant is already in the direction of the abnormal situation.

**Figure 4:**
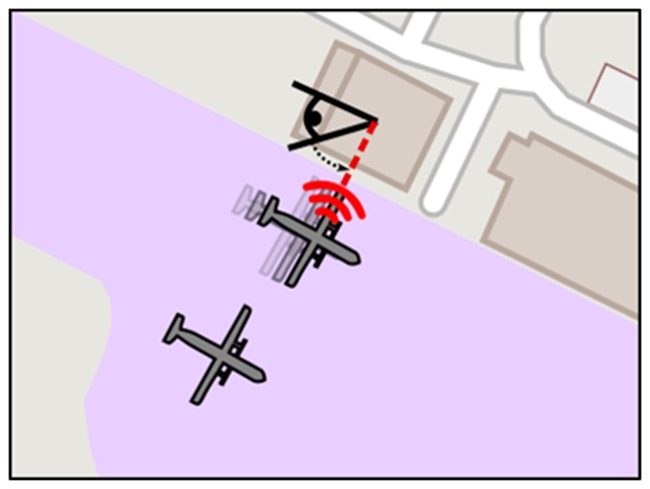
Alert risen by an unauthorized aircraft movement. The alert is spatialized, attracting the gaze of the user in the direction of the abnormal situation.

Spatial sound alert modality is expected to reduce the time taken by the participants to resolve an abnormal situation (e.g., an aircraft moving on parking area without any authorization, Figure 4).

#### Runway Incursion Alert

A runway incursion event can be at the origin of the worst case of incident: a collision with at least one vehicle with an important speed. The related event was here called “RWY.” To support the ATCO in mitigating this risk, a highly disruptive alert has been designed. This alert combines Spatial sound alert (see previous paragraph) and a vibrotactile feedback. The combination of the vibrotactile input with a Spatial sound alert aims at giving cues concerning the event location. The vibrotactile feedback is calibrated to be clearly more highlighted compared to spatial sound provided by Spatial sound alert modality. When an aircraft is in its final approach segment, all other aircraft being or entering on the runway (by crossing the holding point) will trigger the runway incursion alert. The alert is provided with a continuous vibration through the transducer located under the seat of the chair, made of an uncomfortable signal (i.e., modulated in frequency and amplitude). This signal does not allow any habituations. Moreover, to inform the participant of the localization of the incursion, the runway incursion’s specific alert is completed by the generic Spatial sound alert, which is spatialized toward the holding point.

**Figure 5:**
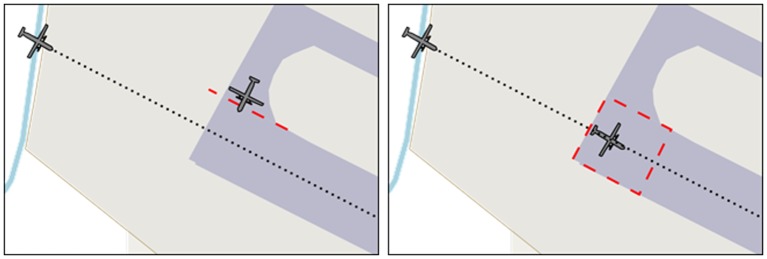
Left: a situation in which the aircraft located on the ground is stopped behind the holding point, generating no runway incursion situation. Right: the aircraft located on the ground entered the runway by crossing the holding; because of its location on the runway while another aircraft is about to land (on the final approach segment), this situation is a runway incursion.

As for Spatial sound alert modality, Runway Incursion modality is expected to reduce the time taken by the participants to resolve a Runway Incursion situation (Figure 5).

### Experimental Subjects

Sixteen professional ATCOs have been involved in the study. Each subject was asked to perform a specific training before starting with the experimental protocol, to get familiar with the simulation platform and employed technologies, and to avoid any learning effect during the scenario’s execution. If the participant told us that he or she was not confident with experimental modalities, the training phase was repeated again. The group was composed of eight males and eight females, with a mean age of 39.4 years (*SD* = 7). None of them reported auditory problem (they are frequently checked due with their profession). Their main experience in control tower was 10 years (*SD* = 6.8). Each participant signed a consent form before the experiment. The experiment was conducted following the principles outlined in the Declaration of Helsinki of 1975, as revised in 2000. It received the favorable opinion from the local Ethical Committee.

### Experimental Protocol

For each ATCO attending the protocol, EEG and ECG recordings were carried out using 13 Ag/AgCl passive wet electrodes (EEG, in particular FPz, AFz, AF3, AF4, Fz, F3, F4, Pz, P3, P4, POz, PO3, PO4) referenced to both the earlobes and grounded to the left mastoid, according to the 10–20 standard (Jurcak et al., 2007) + 1 bipolar channel (ECG) placed on the chest of the subject. The device adopted in the experiment was the BEMicro (from EBNeuro Company) and the sampling rate was set to 256 Hz. In addition, GSR activity was monitored by means of the NeXus-10MKII system (MindMedia BV, Netherlands) and its dedicated software BioTrace+ with a sampling rate of 64 Hz. Biosignals have been synchronized with all the events coming from the simulation platform thanks to a specific device (Trigger Station, BrainTrends srl). In particular, at the beginning of each experimental condition, the simulation platform sent a specific trigger to both the two biosignal amplifiers, in order to be able to synchronize all the information offline.

Once all the biosensors were placed and before starting with the experimental scenarios execution, each subject was asked to perform short recordings, to be used as calibration for neurophysiological indexes computation. In particular, 1 min with eyes closed, to compute the Individual Alpha Frequency (IAF, Klimesch, 1999, see “Neurophysiological Measures” section for further details), and two recordings of 3 min each, of EASY and HARD difficulty levels scenarios, to be used as a calibration for the algorithm used to evaluate the EEG-based workload index (W_EEG_, Aricò et al., 2016b, see “Neurophysiological Measures” section for further details). After these calibration recordings, the two experimental scenarios were run in a random order and the EEG, ECG and GSR signals have been recorded simultaneously during their execution. During the execution of the scenarios, a Subject Matter Expert (SME) provided his subjective judgment about participants’ performance and mental workload. Immediately after each scenario, participants were asked to fill a specific questionnaire to evaluate their perceived mental workload [i.e., NASA Task Load Index (NASA-TLX; Hart and Staveland, 1988; Hart, 2006)].

### Employed Measures

Subjective, behavioral and neurophysiological measures have been employed to compare the two operational solutions (i.e., norm-RT and augm-RT). Table 1 shows a summary of the used measures.

**Table 1:** Measures employed to compare the two operational solutions (i.e., norm-RT and augm-RT).

	Subjective measures	Behavioral measures	Neurophysiological measures
Mental workload	NASA-TLX		W_EEG_ index
Performance	SME evaluation	Reaction time
Arousal			Skin conductance level

### Subjective Measures

#### Cognitive Workload (NASA-Task Load Index)

The questionnaire used to estimate the mental workload was the NASA-TLX, that uses six 100-points range subscales to assess mental workload:

1. Mental demand: *How much mental and perceptual activity was required? Was the task easy or demanding, simple or complex?*
2. Physical demand: *How much physical activity was required? Was the task easy or demanding, slack or strenuous?*
3. Temporal demand: *How much time pressure did you feel due to the pace at which the tasks or task elements occurred? Was the pace slow or rapid?*
4. Performance: *How successful were you in performing the task? How satisfied were you with your performance?*
5. Effort: *How hard did you have to work (mentally and physically) to accomplish your level of performance?*
6. Frustration: *How irritated, stresses, and annoyed vs. content, relaxed, and complacent did you feel during the task?*

The six individual dimension ratings were linearly combined into a global score, ranging from 0 to 100.

#### Subject Matter Expert (SME) Evaluation

Direct and non-intrusive evaluation of ATCOs performance and experienced workload during the execution of the two operational scenarios with the augmented solutions activated (augm-RT) or not (norm-RT), was carried out by the SME, a professional ATCO with more than 25 years of experience in control tower and as ATM instructor.

The SME questionnaire consisted of two questions dealing with the overall performance achieved and workload experienced by the participants. For each question, the SME had to provide a rate from 0 (Very Low) to 100 (Very High). In particular, the SME was asked to evaluate from one side the performance execution of the experimental subjects, as could he act like an instructor. On the other side, the SME should evaluate the workload level that in his opinion the ATCO had experienced overall in that specific scenario.

### Behavioral Measures

Behavioral measurements (i.e., response times) were collected by automatically retrieving response times every time a specific operational events of interest occurred (i.e., SPATIAL or RWY events). With the goal to be able to compare these measurements during the behavioral data analysis, they were acquired during norm-RT *and* augm-RT scenarios. The only difference was that during norm-RT scenario, the participant was not helped with augmentation modalities because they were not activated, but SPATIAL and RWY events were still raised-up. Timers were used to retrieve response time (in milliseconds) for the two types of events:

- For SPATIAL event, related to Spatial sound alert modality: the timer was started when the related unauthorized movement on ground was initiated by the pseudo-pilots, and stopped when the participant successfully managed the induced situation;
- For the RWY event, related to Runway incursion alert modality, the timer was started until the moment that an aircraft entered the runway by crossing the holding point while another one was about to land very shortly. The timer was then stopped when the participant managed the dangerous situation by asking the pilot in short final to go for a go-around procedure.

This was true for norm-RT and augm-RT, but during augm-RT, specifically, the timer was started when the alarm started to sound (and for RWY event, also when the chair started to vibrate), and stopped when the alarm stopped (i.e., when the participant has aligned his head with the azimuth of the event).

Since, at the end, that more than one measurements were collected for each type of event, response times were averaged for each participant and scenario. Unfortunately, the simulation interface that the operators had to interact with, was not fully accessible, then it was not possible to record other kind of behavioral measures apart ascribed reaction times. Anyhow, Reaction Times represent for sure a precious information (one of the most important ones), especially for the comparison of the proposed augmented solutions. In fact, from an operational point of view, it is very important being able to be aware as quickly as possible about possible emergencies, or abnormal situation. Anyhow, we tried to compensate the lack or more behavioral measures by involving the SME and by asking him to rate this kind of overall performance measure.

### Neurophysiological Measures

For each subject we recorded EEG, ECG and GSR activity. In particular, EEG has been used to calculate W_EEG_ index, tonic component of GSR signal was computed to quantify the level of arousal of the operator across the operational scenarios. Heart Rate estimation from ECG signal, and Blink Rate estimation from FPz channel, together with Frontal EEG Theta and Parietal EEG Alpha bands have been estimated to confirm that the two EASY and HARD calibration scenarios were actually able to induce two different workload levels.

#### Heart Rate Estimation

The ECG signal of the EASY and HARD conditions was first filtered by using a 5th-order Butterworth band-pass filter (High-Pass filter: cut-off frequency fc = 5 Hz; Low-Pass filter: cut-off frequency fc = 20 Hz), in order to reject the continuous component and the high frequency interferences, such as that one related to the mains power source. At the same time, such filtering aims to emphasize the QRS process of the ECG signal. The following step consisted in measuring the distance between consecutive R-waves’ peaks (RR distance) of the ECG signal (each R peak correspond to a heartbeat) in order to estimate the HR signal. In this regard, the Pan-Tompkins algorithm (Pan and Tompkins, 1985) has been employed, since it is the most used algorithm for the HR estimation for the ECG signal.

#### Blink Rate Estimation

The blink detection has been performed using a variant of BLINKER pipeline (Kleifges et al., 2017). In its original version, BLINKER algorithm selects the best channel among an arbitrary number of EEG channels, allowing the optimal identification of blinks. However, we forced the BLINKER algorithm to use the FPz channel to detect the blinks. To proceed to the blink detection, the FPz signal has been bandpass filtered between 1 and 20 Hz. Potential blinks have amplitude 1.5, the standard deviation of the signal, duration higher than 100 ms and interblink interval higher than 50 ms. Among these only the blinks showing a correlation with the tent-like shape higher than 0.9 have been considered. The last check selects the blinks with Positive Amplitude Ratio higher than 3 to remove saccades. For each condition, it has been computed the number of Blinks per minute (that represents the Blink Rate).

#### EEG Spectral Features Estimation and W_EEG_ Index Evaluation

**Figure 6:**
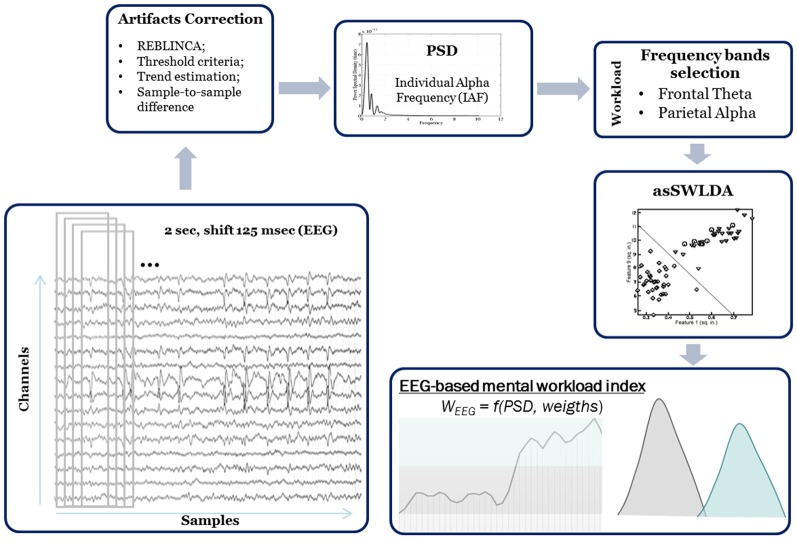
Electroencephalography (EEG) processing chain for the W_EEG_ index computation.

As stated before, depending on the literature evidence, theta EEG rhythms over frontal sites, and alpha EEG rhythms over parietal sites have been taken into account for the computation of the EEG-based workload measure, i.e., the W_EEG_ index. The W_EEG_ index has already been validated in many operational settings, in which it was used to assess the workload experienced by the subjects across different difficulty levels. The W_EEG_ index is based on the application of a linear regression algorithm, the *automatic stop StepWise Linear Discriminant Analysis* (asSWLDA; Aricò et al., 2016b), able to extract EEG features sensitive to workload assessment (i.e., Frontal Theta Bands and Alpha Parietal Band). In the following, all the algorithm steps that allow to compute the W_EEG_ index starting from the EEG signal have been reported (Figure 6). Different processing and artifact removing algorithms have been applied to the EEG signal in order to: (i) remove eye-blinks contribution, that could affect frequency bands related to workload and consequently introduce a bias in the measure; and (ii) remove all the other sources of artifacts (i.e., muscular artifact, saturation of amplifier). First of all, the EEG channels have been first band-pass filtered with a 5th-order Butterworth filter [low-pass filter cut-off frequency: 30 (Hz), high-pass filter cut-off frequency: 1 (Hz)]. The FPz channel has been used to remove eyes-blink artifacts from the EEG data by using the regression-based algorithm REBLINCA (Di Flumeri et al., 2016), that could affect the EEG frequency bands involved in workload assessment. With respect to other regressive algorithms (e.g., Gratton method, Gratton et al., 1983) the REBLINCA algorithm has the advantages to preserve EEG information in blink-free signal segments by using a specific threshold criterion that recognizes the occurrence of an eye-blink, and only in this case the method cleans the EEG signals. If there is not any blink, the method has not any effect on the EEG signal. The band-pass filtered [1–7 (Hz), 5th order Butterworth filter] FPz signal has then been used as template to remove eye-blinks contribution from the EEG signal. At this point, following the algorithm described in Aricò et al. (2016b), the EEG signals have been segmented into epochs of 2 s, shifted of 0.125 s. Specific procedures of the EEGLAB toolbox have been used (Delorme and Makeig, 2004) to remove artifacts generated by muscular activity and bioamplifier saturation. In particular, three criteria have been applied. *Threshold criterion*: if the EEG signal amplitude exceeds ±100 (μV), the corresponding epoch would be marked as artifact. *Trend criterion*: each EEG epoch has been interpolated in order to check the slope of the trend within the considered epoch. If such slope was higher than 10 (μV/s) the considered epoch would be marked as artifact. *Sample-to-sample difference criterion*: if the amplitude difference between consecutive EEG samples was higher than 25 (μV), it meant that an abrupt variation (no-physiological) happened and the EEG epoch would be marked as artifact. At the end, all the EEG epochs marked as artifact have been rejected from the EEG dataset with the aim to have an artifact-free EEG signal from which estimate the brain variations along the different conditions. All the previous mentioned numeric values have been chosen following the guidelines reported in Delorme and Makeig (2004).

At this point, *Power Spectral Density* (PSD) was calculated for each EEG epoch using a Hanning window of the same length of the considered epoch (2 s length, that means 0.5 Hz of frequency resolution). Then, the EEG frequency bands of interest have been defined for each subject by the estimation of the *IAF* value (Klimesch, 1999). In order to have a precise estimation of the alpha peak and, hence of the IAF, as stated before the subjects have been asked to keep the eyes closed for a minute before starting with the experiments. Finally, a spectral features matrix (EEG channels × Frequency bins) has been obtained in the frequency bands directly correlated to the mental workload. In particular, only the theta rhythm (IAF-6 ÷ IAF-2), over the EEG frontal channels (AFz, AF3, AF4, Fz, F3, F4), and the alpha rhythm (IAF-2 ÷ IAF+2), over the EEG parietal channels (Pz, P3, P4, POz, PO3 and PO4) have been considered as variables for the mental workload evaluation.

At this point, a linear classification algorithm (asSWLDA, Aricò et al., 2016b), an upgraded implementation of the well-known SWLDA algorithm has been used to select the subjective discriminant EEG spectral features related to the workload. With respect to the standard SWLDA approach, the asSWLDA algorithm embeds an automatic procedure to select the best number of relevant features to keep into the discrimination model. This property was demonstrated so far to increase the robustness to both the under and the overfitting phenomenon (Aricò et al., 2016b). Once trained with specific “calibration data,” the algorithm can be used to compute a workload index (i.e., W_EEG_ index) on other data (i.e., experimental scenarios) by combining the selected EEG features with specific weights in output from the model itself. In particular, the algorithm has been calibrated by using the EASY and HARD related data already described.

In conclusion, *z*-score transformation (Zhang et al., 1999) has been used to compute a normalization of W_EEG_ index distribution.

#### Skin Conductance Level Estimation

The signal related to the Skin Conductance, named hereafter GSR, has been recorded with a sampling frequency of 64 Hz. A constant potential was applied in order to induce a skin electrical current. The variations of such current are a function of the skin conductance variations. The recorded signal was then entirely processed by using the Matworks MATLAB software. First of all, the signal was down-sampled to 16 Hz, in order to reduce the data amount. Also, the signal was then filtered through a 5th order Butterworth low-pass filter (cut-off frequency = 2 Hz) in order to remove all the higher frequency components that are not related to skin sweating activity. Finally, the signal was processed by using the Ledalab suite, a specific open source toolbox implemented within MATLAB for the GSR processing. The Continuous Decomposition Analysis (Benedek and Kaernbach, 2010) has been applied in order to separate the Tonic (Skin Conductance Level—SCL) and the Phasic (Skin Conductance Response—SCR) components of the GSR. In the following analysis, the mean value of the SCL during the experimental scenarios has been taken into account.

### Statistical Analysis

All the statistical comparisons have been performed through two-sided Wilcoxon signed-rank tests. In fact, data come from multiple observations on the same subjects, but it is not possible to assume or robustly assess that the observations distribution is Gaussian, therefore paired non-parametric tests have been used (Siegel, 1956).

## Results

### Calibration Conditions Analysis

**Figure 7:**
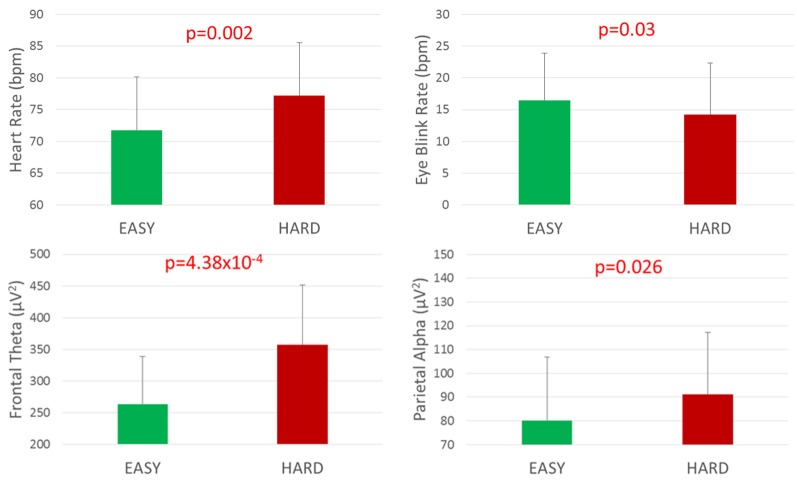
Neurophysiological measures between EASY and HARD conditions. Significant *p*-values have been marked in red. Values showed are mean and standard error, at a confidence interval of 0.95.

HR, Blink Rate, Frontal Theta and Parietal Alpha EEG bands values have been calculated on the data related to calibration conditions (EASY and HARD) in order to confirm that they were well designed (i.e., were able to induce two different workload levels, Figure 7). Statistical results suggested that all the considered neurophysiological indicators changed significantly among EASY and HARD conditions, confirming the suitability of the design of the EASY and HARD conditions in inducing two different levels of experienced workload. In particular, HR increased significantly (EASY = 72 ± 8; HARD = 77 ± 8; *p* = 0.002) and the Eye blink rate decreasing significantly (EASY = 16 ± 7; HARD = 14 ± 8; *p* = 0.03) during the HARD condition. Regarding EEG frequency bands, Frontal Theta significantly increased (EASY = 264 ± 75; HARD = 357 ± 94; *p* = 4.38 × 10^−4^), and even Parietal Alpha increased (EASY = 80 ± 27; HARD = 91 ± 26; *p* = 0.013) with increasing difficulty level (HARD).

### Subjective

The perceived workload was evaluated considering both the NASA-TLX workload score (i.e., directly filled from experimental subjects, W_NASA-TLX_), and post-run SME assessment (W_SME_).

**Figure 8:**
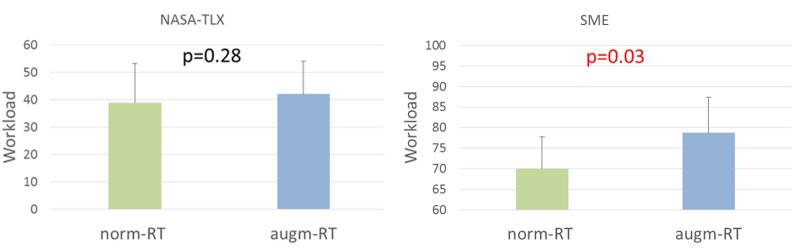
Subjective post-run workload scores assessed by experimental subjects (i.e., W_NASA-TLX_, on the left) and subject matter expert (SME; i.e., W_SME_, on the right) values for each condition. Significant *p*-values have been marked in red. Values showed are mean and standard deviation.

Statistical test exhibited no significant trends between the two conditions (norm-RT = 39 ± 14 and augm-RT = 42 ± 12, *p* = 0.28). On the contrary, scores provided by the SME highlighted a significant increase in overall workload during the augm-RT (70 ± 10) condition with respect to the norm-RT (79 ± 9) one (*p* = 0.03, Figure 8).

**Figure 9:**
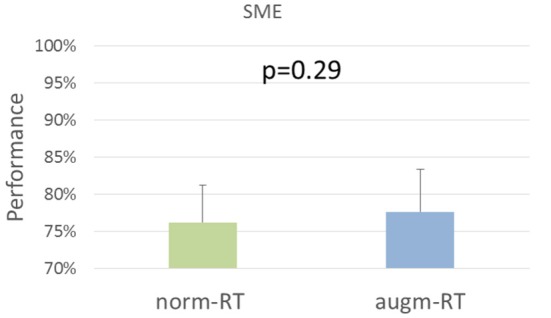
Performance values assessed by the SME for each experimental condition. Values showed are mean and standard deviation.

Even Performance values provided by the SME did not highlight any significant difference between the two experimental conditions (norm-RT = 76 ± 5; augm-RT = 78 ± 6; *p* = 0.29, Figure 9).

### Neurophysiological Results

#### EEG-Based Workload Index (W_EEG_) Evaluation

**Figure 10:**
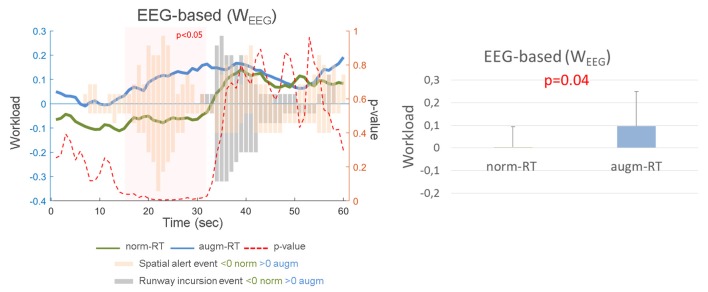
W_EEG_ score exhibited a significant increase in the experienced workload during the augm-RT condition with respect to the norm-RT one, which is consistent with the results provided by the SME workload assessment. On the left side it was reported a grand average of the W_EEG_ index over all the subjects, for each experimental scenario. We reported the events distribution for all the subjects, for the whole time duration (orange Spatial Alert events, and gray Runway incursion ones). Bars higher than 0 are referred to augm-RT scenario events, while bars lower than 0 are referred to norm-RT scenario events. It has also been reported in red the point by point *p*-value, highlighting that the significance was reached (i.e., red transparent rectangle) just around the appearance of the spatial sound events. On the contrary, during the appearance of Runway incursion events (i.e., gray bars), the workload increased in both the scenarios, in fact statistics did not show any significant trend. On the right it has been reported the mean and standard deviation of W_EEG_ index related to the two scenarios.

EEG features (i.e., Frontal Theta and Parietal Alpha) of EASY and HARD conditions have then been used to calibrate the asSWLDA algorithm for each subject, in order to compute the W_EEG_ index for each experimental scenario (i.e., norm-RT and augm-RT). Statistical analysis showed a significant overall increase (norm-RT = 0.004 ± 0.09; augm-RT = 0.1 ± 0.15; *p* = 0.04) of the index during the augm-RT scenario execution with respect to the norm-RT one (Figure 10, right). In addition, we performed a point by point statistical analysis (two-sided Wilcoxon signed-rank tests) between the W_EEG_ indexes of each scenario to highlight the reason of this increase in experienced workload. The test highlighted that the significance was reached (i.e., red rectangle) just around the appearance of the spatial sound events (Figure 10, left). On the contrary, during the appearance of Runway incursion events (i.e., gray bars), the workload increased in both the scenarios, in fact statistics did not show any significant trend. In this regard, we reported in Figure 10 both the W_EEG_ indexes of the two scenarios second by second. In addition, we reported *p*-values for each time point, and the events distribution for all the subjects.

#### Skin Conductance Level

We computed the SCL of the GSR signal for each experimental scenario, in order to investigate the emotional involvement (i.e., arousal level) of operators during the execution of the two scenarios.

**Figure 11:**
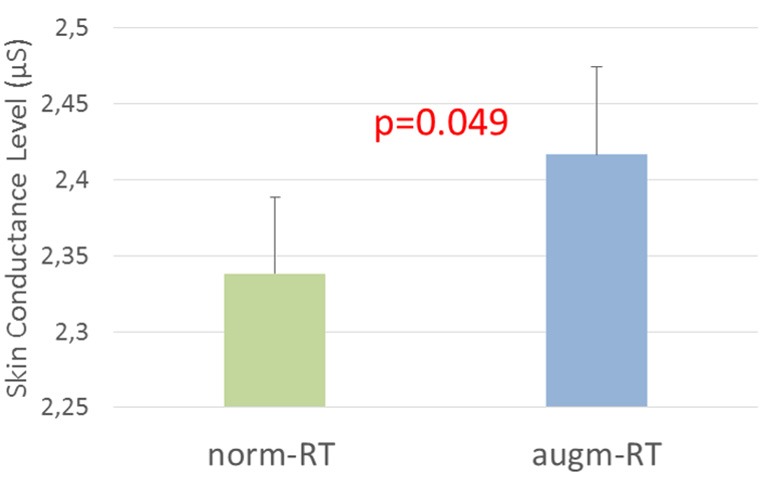
The analysis of Skin Conductance Level (SCL) of the galvanic skin response (GSR) signal revealed an increase in the arousal during the augm-RT condition with respect to the norm-RT one. Significant *p*-values have been marked in red. Values showed are mean and standard deviation.

The statistical analysis showed a significant increase (norm-RT = 2.34 ± 0.84; augm-RT = 2.42 ± 0.82; *p* = 0.049) of the Tonic component during the augm-RT scenario execution with respect to the norm-RT one (Figure 11).

### Single Event Analysis

The simulation platform developed at ENAC allowed to record the reaction time needed to the experimental subjects to reply to specific events under which the augmented solutions could be activated (i.e., augm-RT scenario) or not (i.e., norm-RT scenario). In the following, the reaction times and the related W_EEG_ index recorded during these events (i.e., starting from the onset of the event, until the ATCO addressed the issues highlighted within the specific event) have been analyzed.

#### Behavioral Results

**Figure 12:**
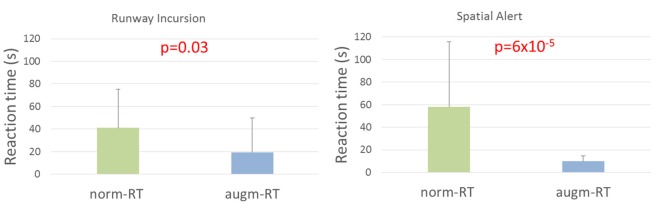
Reaction times recorded by the simulation interface exhibited a significant decrease of the time needed by the subjects to identify and react to the specific event, highlighting a local increase in performance induced by the specific augmented solutions. Significant *p*-values have been marked in red. Values showed are mean and standard deviation.

Statistical results highlighted a significant decrease in reaction times needed from the experimental subjects to identify (and react) to the specific events (Runway Incursion norm-RT = 41 ± 35; augm-RT = 20 ± 30; *p* = 0.03; Spatial Alert norm-RT = 58 ± 57; augm-RT = 10 ± 5; *p* = 6 × 10^−5^) if the augmented solutions were activated (i.e., augm-RT) in comparison to the scenarios without any augmented solution activated (norm-RT, Figure 12).

#### W_EEG_ Scores

**Figure 13:**
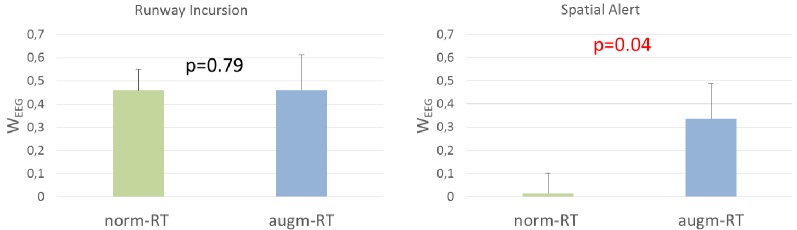
W_EEG_ values calculated during the specific events highlighted no significant difference during the Runway incursion event, and a significant increase during the Spatial Alert event. Significant *p*-values have been marked in red. Values showed are mean and standard deviation.

Thanks to the possibility to calculate the EEG-based workload index along the execution of the two scenarios, over time, it was possible to measure the experienced workload of the subject during the occurrence of the specific operational events where augmentation could (augm-RT) or not (norm-RT) be applied. Statistical analysis did not highlight any statistical difference in the workload experienced by the experimental subjects during the events related to Runway incursion alert (norm-RT = 0.46 ± 0.03; augm-RT = 0.46 ± 0.06; *p* = 0.79). In addition, if we consider norm-RT scenario from an operational point of view, it can be seen that Runway incursion event induced a significant higher workload than the Spatial alert event (1.7 × 10^−6^). On the contrary, during the event related to Spatial sound alert, it was highlighted with a significant increase in the experienced workload (norm-RT = 0.01 ± 0.28; augm-RT = 0.34 ± 0.54; *p* = 0.04) if the augmented solutions were activated, with respect to the condition without any augmentation activated (Figure 13).

**Figure 14:**
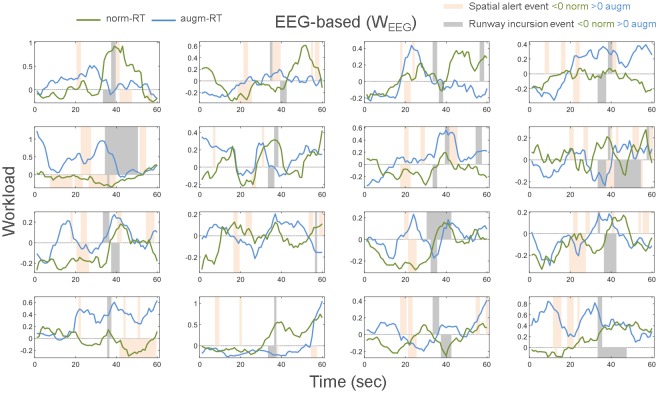
W_EEG_ index shape for each of the 16 experimental subjects and related operational events appearance (i.e., Spatial Alert and Runway Incursion) have been reported. In particular, time duration of each event has been reported for each of the two scenarios (i.e., norm-RT and augm-RT). Bars higher than 0 are referred to augm-RT scenario events, while bars lower than 0 are referred to norm-RT scenario events.

Figure 14 shows for each of the 16 experimental subjects involved in the study-specific W_EEG_ behavior second by second and related events appearance.

## Discussion

The present work aimed to demonstrate that the combination of information coming from neurophysiological measures, together with behavioral ones, could provide a more comprehensive assessment in the design process of new technologies and solutions in operational environments with respect to the evaluation of only behavioral and/or subjective measures. In this regard, the unimodal assessment could not be enough informative to investigate if a new technology could be effective or not in order to enhance the operator performance. In fact, considering the subjective measures alone, an incongruence emerges: the NASA-TLX could suggest that the two solutions did not induce any decrease in perceived workload. On the other side, the SME glimpsed an overall increase in the operator workload, but any improvement in the overall performance. This point highlights also the low resolution of the NASA-TLX questionnaire in assessing perceived workload, as demonstrated in de Waard and Lewis-Evans (2014). In this regard, taking a look to neurophysiological measures, in particular to the W_EEG_ index, it highlights an increase in the experienced cognitive workload if the augmented solutions were activated (consistently with the SME workload assessment), and in particular, a statistically significant increase during one specific event (i.e., the one related to Spatial sound alert). On the contrary, during the Runway incursion event both the solutions (norm and augm-RT) induced a similar increase in workload, and no differences were highlighted between the two modalities in this case. Anyhow, this information alone could not be enough if we do not look at the same time at the performance achieved by the experimental subjects. In this regard, the overall performance filled by the SME did not reveal any difference between the two experimental conditions (i.e., norm-RT and augm-RT), but subject’s reaction times significantly decreased during the experimental events (RWY and SPATIAL) if the related augmented solutions were activated. If we consider also the significant increase in the tonic component of the GSR signal during the augm-RT related scenario, that implies a higher psychophysiological involvement (i.e., arousal) of the experimental subjects. Therefore, the overall story can suggest us the following insights:

1. The proposed augmented solutions induced a local increase in subject performance on those operational events where the augmentation was applied. The overall performance seems to not be affected by the proposed solutions.
2. The augmented solutions induced an increase in the workload experienced by the participants. Anyhow, this increase is still acceptable and also fruitful, since it did not negatively impact the performance (that indeed locally increased) and has to be intended only as a consequence of the higher engagement of the controller. This behavioral effect is totally in line with physiological results obtained in terms of arousal (following point).
3. Augmented solutions induced an increase in the arousal level of the subjects, representing a positive aspect for the overall performance assessment. This result corroborates the workload highlights and supports the overall interpretation of the propaedeutic effect of augmented solutions in enhancing the controller engagement, and consequently its performance. In fact, according to the inverted U-shape relationship theory between human performance and psychophysiological activation (conjugated with the various concepts of arousal, stress, workload; Janelle, 2002), in general, a proper increase in workload and arousal is not disruptive, but necessary for humans to achieve their best performance.

Finally, taking again a deeper look at the single event assessment, it is clear that augmented solutions induce a decrease in the time needed by the operator to catch the emergency and react to it. In other words, although the overall performance level of the operator seemed to not change significantly, the augmented solutions induced a local increase in performance. Anyhow, the related events during the augm-RT scenario, in particular during the event related to the Spatial sound alert, induced a higher experienced workload, identified by the W_EEG_ index. This effect could be due to the information that the ATCOs would not be aware of, if they would not have been alerted. Even if in this specific experiment workload increase did not induce a decrement in performance, it has to be stressed on the possible negative effects of increased workload over time, such as fatigue. Increased workload and resulting fatigue have in fact continued to play a major role in many transport accidents to the present day (European Agency for Safety and Health at Work, 2011). In this regard, training of operators could have a significant effect on the experienced workload. Even if the use of the two sensory channels of hearing and touch could be seen as natural, the novelty for them of these kind of interaction techniques and the information provided consequently could need a longer appropriation time, implying specific care in learning sessions. In other words, although ATCOs have been well trained to use in a correct way the proposed augmented solutions before the experiment and they could be useful in specific operational situations (i.e., Runway Incursion and Spatial sound alert), they could become in general too much distracting, inducing in some way an increase in experienced workload. However, novelty and lack of familiarity can be modulated by learning, which could mitigate this effect in the long term by decreasing workload, and eventually related induced fatigue effects or potential lack in performance.

### Highlights for Multimodal Interaction in Remote Tower

The present work allowed to provide few suggestions about the implementation of multi-modal interaction in future remote towers platforms. In particular:

- **Auditory and Vibrotactile feedback shall be used to improve the salience of relevant remote operational events or highlight changes of operational status**. The outcomes of this experiment highlighted a possible advantage of implemented Augmented solutions when applied on specific operational events (i.e., Spatial Alert and Runway Incursion Alert), since the related reaction time values were significantly shortened once Augmented solutions were activated.
- **Multimodal remote tower platform implementation shall take into account ATCO control tower soundproofing background**. Results showed that providing ATCOs with visual, audio and vibrotactile feedback seemed to increase the ATCOs experienced workload level. ATCO feedback collected during the validation activities suggest that this effect may be influenced by the controller’s familiarity with specific tower environment; namely if ATCO used to work in a soundproofed tower or not. The constant provision of stimuli that are not necessarily associated to relevant operational events, seems to be perceived as disturbing in a control tower soundproofed from the outside aerodrome environment. The provision of multimodal feedback in a multimodal remote tower platform shall be modulated according to ATCO familiarity soundproofed tower environment; in order to minimize the distracting effect that may induce a decrease in performance and an increase in experienced workload.
- **The implementation of augmented multimodal feedback in remote tower requires an extended familiarization period**. Results showed an increase of cognitive workload while using augmented solutions because the novelty of the information and/or by the novelty of the modalities used (e.g., audio and vibration). Nowadays, ATCOs use the audio and haptic modalities unconsciously since many control towers are sound proof, and vibration is generally not felt. ATCOs will need a more intensive training and familiarization period to understand the real effect of the provided augmentations on performance.”

## Conclusion

The present work demonstrated the suitability of neurophysiological measures during the design and validation phase of new solutions/technologies/tools in operational environments, in particular for remote tower operations. More specifically:

- The computed W_EEG_ index can be used for the evaluation of mental workload in operational settings and could provide a measure at a higher resolution if compared with the classical assessing methods (i.e., questionnaires). In this regard, it is able to provide information over time (i.e., at a level of few seconds), opening up the possibility to assess the workload level experienced by the operators during specific operational activities or particular events, allowing to identify critical points during the operational activity or along the design process and to optimize the human-machine interaction, even in real-time. As an example highlighted in this study, the W_EEG_ index revealed that the Runway incursion events induced significantly higher workload than Spatial alert events. By using subjective measures it was not possible to highlight this kind of behavior, and this information could instead be relevant in operational activities.
- GSR was confirmed to be quite sensitive in showing the psychophysiological activation of subjects during operational activities, therefore appearing as a powerful and less invasive tool for evaluating human engagement.
- The evaluation of neurophysiological measure is able to provide additional information with respect to behavioral and subjective measures, and its employment during the pre-operational activities (e.g., design process) could allow a more holistic and accurate assessment of new technology/solutions.

## Data Availability

The datasets for this study will not be made publicly available because Data were collected during an European project, so the dataset are property of the consortium. Anyhow, excel with aggregated data related to this manuscript can be provided if requested.

## Ethics Statement

This study was carried out in accordance with the recommendations of “CERNI: Comité d’Ethique sur les Recherches NonInterventionnelles” with written informed consent from all subjects. All subjects gave written informed consent in accordance with the Declaration of Helsinki. The protocol was approved by the “Comité d’Ethique sur les Recherches NonInterventionnelles.”

## Author Contributions

PA, GD, GB and NS: biosignals recordings, data analysis and article writing. MR: behavioral data analysis, and article writing. J-PI and CH: design and development of the simulation platform. MT, AF and SP: experimental design, experiments execution and article check. VB and MM: biosignal data analysis and article check. AT: experimental design and article check. FB: experimental protocol designing and article check.

## Conflict of Interest Statement

PA, GD, GB and NS are employed by company BrainSigns srl. MT, AF and SP are employed by company DeepBlue srl. MM is employed by company BrainTrends srl.
